# Modern Monetary Theory: The Right Compass for Decision-Making

**DOI:** 10.1007/s10272-022-1041-x

**Published:** 2022-04-08

**Authors:** Dirk Ehnst

**Affiliations:** TU Chemnitz, Thüringer Weg 7, Raum 324, 09126 Chemnitz, Germany

**Keywords:** E12, B52, B4

## Abstract

In the November/December 2021 issue of Intereconomics, Françoise Drumetz and Christian Pfister examine Modern Monetary Theory (MMT) and approach it from the policy consequences that would follow. This paper is a reply to Drumetz and Pfister. It restates the core of MMT and offers some suggestions for central banks. Theories are explanations of what we see, and MMT describes money creation and destruction. Hence, MMT cannot be and is not a political manifesto. In contrast to most other theories of money, MMT is falsifiable in its core statements, which are based on a balance sheet approach to macroeconomics. Since many central banks already educate the public about the creation of modern money through bank lending, it would be most welcome if they would do the same for the creation of modern money through government spending. Here, MMT and central bankers can find common ground to move forward and leave the theory of loanable funds and that of the money multiplier behind.

Teaching macroeconomics in 2022 is an interesting exercise. The textbooks usually rely on the money multiplier. They assume that the central bank lends to banks, which then lend to households and firms. This is contrasted by the announcements of practitioners, mostly central bankers and bankers.[1] The St. Louis Fed tweeted the following statement to draw attention to Ihrig et al. (2021): "Many econ textbooks include outdated information on how Fed policy influences banks and the economy. Educators should abandon the 'money multiplier,' a popular model that is now obsolete".[2] If, however, the money multiplier is wrong, then what becomes of the discussion of banks as intermediaries, equilibrating saving and investment? The Bundesbank (2017, 17) writes: "This [the stylized example of the creation of money] refutes a popular misconception that banks act simply as intermediaries at the time of lending – i.e., that banks can only grant loans using funds placed with them previously as deposits of other customers". So, macroeconomics is in need of a new theory. The textbook models have fallen apart, and a new theory of money is needed. That theory should be Modern Monetary Theory (MMT), which over the last 25 years has matured into a legitimate school.[3]

As retold by Ehnts (2020, 293), mainstream economists do not believe that "countries that borrow in their own currency should not worry about government deficits because they can always create money to finance their debt". Looking at the result from a survey,[4] not a single economist agreed with that statement. If these economists had been right, we would have seen many governments running out of money in 2020 and 2021. After all, tax revenues collapsed, government spending increased and public deficits and public debts skyrocketed. Surely, the Greek government, surpassing 200% of public debt to GDP in 2021, would be in for a repeat of the euro crisis. It did not happen. As we all know by now, a government cannot run out of its own money for technical reasons.[5] *The Wall Street Journal* recognises that "important elements of MMT are accepted by much of the financial establishment" and that "the lesson of 2020 was that MMT is right" because "a government need never default on

1 One of the first practitioners to stress that households and firms cannot borrow reserves is Sheard (2013).

2 See https://twitter.com/stlouisfed/status/1447612987196456972.

* This article is a reply to Drumetz and Pfister (2021a, 2021b). The author thanks Pavlina Tcherneva and Phil Armstrong for comments on the paper. An earlier and longer version of this paper was published as Ehnts (2022).

**Dirk Ehnts**, Hochschule Magdeburg-Stendal, Germany.

3 JEL codes E12 and B52 include Modern Monetary Theory, Mitchell et al. (2019) published a 600-page textbook that can replace mainstream textbooks and Kelton (2020) is a New York Times bestseller.

4 https://www.igmchicago.org/surveys/modern-monetary-theory/.

5 There is nothing to stop a state from enacting laws that would stop the government spending more, like the US debt ceiling. Then, however, it is a political decision to run out of money. See Ehnts (2020b).

debt issued in its own currency" (Mackintosh, 2021). In the eurozone, all national governments made their payments on time – all of them. This needs to be explained.[6] The recent article by Drumetz and Pfister (2021a), published first as a (longer) Banque de France working paper (Drumetz and Pfister, 2021b), could be the start of a conversation about how to reconstruct macroeconomics and narrow the deep gulf between theory and practice in both monetary theory and macroeconomics.[7]

## Mainstream macroeconomics, MMT and real MMT: Theory and the table

Drumetz and Pfister (2021a, 360) start their examination of the meaning of MMT using a table that summarises their views on both mainstream (theory) and MMT. The table (reproduced as Table 1) seems to be a good starting point for a discussion of their paper. The first column describes the issue discussed, followed by the column that summarises the mainstream view, one that summarises MMT as seen by Drumetz and Pfister (2021a) and one that summarises MMT from my own view. Apart from the issue of unemployment, I differ with the authors' view of MMT. The reason is, I suppose, that the authors approached MMT from the wrong side. Starting with the research question of what the meaning of MMT would be (in the sense of economic policy or institutional reform), they ignored its logical core and failed to recognise the methodological differences from the mainstream approach. This would be comparable to a critique of the mainstream theory by MMT authors that would completely ignore the mathematical model at the core and just discuss the policy implications, i.e. its supposed meaning. This kind of approach implies that the theory is just intellectual hand-waving intended to justify the policy conclusions. But that is not how MMT works.[8]

MMT is, first and foremost, a balance sheet approach to macroeconomics. At its very core lie reserve accounting, then deposit accounting, and then sectoral balances accounting. There is very little behaviour in any of this. Equilibrium rules as all balances balance – in both flows and stocks – and there are no assumptions apart from the existence of a central bank, a Treasury, a banking system and some households and firms. MMT can only be learned by mastering its balance sheet approach. It can only be engaged by discussing the balance sheet operations it puts forward. It is here where value is added. Therefore, I suggest looking at some of these explanations in more detail.

First of all, the main insight of MMT is that the mainstream has the sequence wrong. Whereas they assume that government expenditure is financed by taxes (Table 1, row 1), MMT assumes that government spending is financed by money creation.[9] MMT stresses that the central bank, empowered by the law and serving the state, is the monopoly issuer of currency. In the eurozone, this would be the European Central Bank (ECB) and the national central banks. This logically means that the state has to spend *before* taxes can be paid in euro. When taxpayers pay their taxes (or banks buy government bonds on the primary market), they first need to have state money. "As the sole issuer of euro-denominated central bank money, the Eurosystem will always be able to generate additional liquidity as needed", ECB president Lagarde said according to Reuters (2020).[10] As Kelton (2000) argues, issuers of currency finance their spending by creating money when they spend and cannot do otherwise.[11]

When the ECB buys government bonds or other financial assets in the context of its quantitative easing or its asset purchase programmes, it "increases the price of these bonds and creates money in the banking system", as the ECB (2021) explains on its webpages. With "money" the ECB means "central bank deposits", also called reserves, since it pays with electronic money and not cash. This process is well understood. In an interview with *60 Minutes*, former Federal Reserve Chair Ben Bernanke (2009), was asked where the money the Fed lends would come from. "It's not tax money," Bernanke said, "The banks have accounts with the Fed, much the same

6 The explanations of mainstream economists seem unconvincing. Krugman (2021), for instance, writes: "But is the Fed really financing the budget deficit? Not really. At a fundamental level, households are financing the deficit: the funds being borrowed by the government are coming out of the huge savings undertaken by families saving much of their income in an environment where much of their usual consumption hasn't felt safe." The problem with this is that obviously the Fed does not borrow household savings (or rather saving since this is about flows). It sells sovereign securities to banks only.

7 Some points made in the working paper have been omitted from this version. These points are addressed in Ehnts (2022).

8 MMT started in 1996, when Warren Mosler contacted some academics to discuss monetary theory. Mosler, who worked as a banker, but also constructed racing cars (and a ferry), certainly was not looking to write a political manifesto. In Mosler (1995), he thanks Arthur Laffer for "valuable literary assistance and research with this work". In Mosler (1997), his first peer-reviewed academic journal article, he describes at length how the monetary system works and how we can use it to achieve full employment and price stability – hardly a political manifesto.

9 It is not a coincidence that his view was used by Margaret Thatcher, who claimed that there would only be taxpayers' money and no public money. The opposite is true, but most macroeconomic textbooks do not reflect that.

10 In their abstract, Drumetz and Pfister (2021) point out that MMT would have gained prominence "in the media and in the public". In 2019, Mario Draghi reportedly said that "the ECB should examine new ideas like MMT" (Bloomberg, 2019a) while Christine Lagarde said that "MMT is no panacea but may help fight deflation" (Bloomberg, 2019b). John Yarmouth, chair of the House Budget Committee, defended the Biden administration's policy in terms of MMT, as the *New York Times*' (2021) Peter Coy notes.

11 See Tymoigne (2014) for further details on interactions between the Treasury and the Federal Reserve Bank.

Table 1
**Mainstream view, Drumetz and Pfister MMT view and original MMT view**

| | Mainstream view | Drumetz and Pfister MMT | Original MMT |
|---|---|---|---|
| 1 Government expenditure is financed by... | taxes | issuing currency | it is not "financed" |
| 2 Public debt sustainability... | can be an issue | cannot be an issue | is a political issue (if "debt" in own currency) |
| 3 Public bonds are issued... | to finance the public deficit | to distribute income as part of an interest rate maintenance strategy | as part of an interest rate maintenance strategy and/or to satisfy eurozone rules |
| 4 Access of government to central bank financing... | should be limited | is unlimited | depends on the laws |
| 5 Public debt purchased by the central bank... | should be paid off | is paid off | constititutes an asset swap for banks |
| 6 Crowding out... | can be an issue | cannot be an issue | cannot be the result of a lack of loanable funds |
| 7 Monetary policy... | has a role to play to stabilise the economy | has no role to play to stabilise the economy | has a role to play to stabilise the economy |
| 8 Interest rates... | are a market variable | are set by the government | are set by the central bank |
| 9 Inflation... | is a monetary issue | is a fiscal policy issue | is a complex phenomenon |
| 10 Unemployment... | cannot be fully eliminated | can be fully eliminated | can be fully eliminated |
| 11 Conventional structural policies... | are positive | are negative | can be positive or negative |
| 12 A sovereign economy... | should be competitive | does not have to be competitive | should aim for full employment and price stability |
| 13 Skills... | are important determinants of income | are loosely linked to income | are important determinants of output |
| 14 Social welfare... | has a cost | has no cost | has a cost |

Source: Drumetz and Pfister (2021a); author's own elaboration.

way that you have an account in a commercial bank. So, to lend to a bank, we simply use the computer to mark up the size of the account that they have at the Fed." These are the changes in the respective balance sheets when the Fed extends a loan to a bank that has enough collateral.

It is obvious who is the issuer of currency and who is the user of currency. The Fed creates reserves when it spends or lends. Reserves are created by the computer software that the Fed runs – the payment system. The central bank is the score keeper of its society.[12] This is just how commercial banks work. These create bank deposits when lending, which are destroyed at repayment (McLeay et al., 2014).

To understand questions concerning public debt and fiscal sustainability, we need to look at the way a national (federal) government spends. It is at the level of balance sheets, which are descriptions of reality, that we can expect to find an answer. The following description of the federal government of Germany spending €100 is based on Ehnts (2016, 119).[13] We assume that the day has just started and that the Treasury account of the federal government of Germany (*Zentralkonto des Bundes*) stands at zero. The Treasury now instructs the German central bank to execute a payment of €100 to a household, who has supplied the Treasury with goods and services. The Bundesbank accordingly credits the account of the seller's bank, which then credits the account of the seller. At the same time, forced by its rules of operation, the Bundesbank debits the Treasury account. Table 2 shows what the balance sheets look like.

If this is how a federal government spends in the eurozone, there is no possibility that it can "finance" its spending. Its central bank always creates new reserves when it spends on behalf of the government. It cannot spend tax revenues or bond revenues. As the name implies (from French *revenir*, to come back), when taxes or bonds are paid, the government's money comes back (revenue) to the government. There is one complication, though. In the eurozone, central banks are not allowed to finance their governments. This is why at the end of the day the Treas-

12 See Armstrong and Mosler (2019).

13 A more recent version can be found in Ehnts (2020a).

Table 2
**Government spending of the German federal government**

| Deutsche Bundesbank | | | |
|---|---|---|---|
| | | Reserves | €100 |
| | | Treasury account | -€100 |

| Treasury | | | |
|---|---|---|---|
| Treasury account | -€100 | Net wealth (Δ public debt) | -€100 |

| Bank | | | |
|---|---|---|---|
| Reserves | €100 | Deposits | €100 |

| Household | | | |
|---|---|---|---|
| Deposits | €100 | Net wealth | €100 |

Source: Ehnts (2016).

ury account has to go back to zero. This can be achieved by booking tax and bond sale revenues to the account. The balance in the Treasury account is not money, as the Bundesbank itself notes.[14] It is a number that matters for operational reasons. The Bundesbank can only spend for and on behalf of the Treasury if at the start of the day the Treasury's account is non-negative. Tax and bond revenues are not about financing, but about creating a green traffic light for the Bundesbank. This is a political complication that other monetary systems do not have. So, in the eurozone a national (federal) government cannot run out of money as long as:

1. tax revenues are high enough to bring the Treasury account back to zero or
2. bond revenues are high enough to bring the Treasury account back to zero or
3. tax and bond revenues together are high enough to bring the Treasury account back to zero.

This means that a eurozone national government does not run out of money until it has exhausted its tax revenues and bond revenues. It would only run out of money due to political reasons that are hardwired into the laws of the European Union, not because its central bank cannot create more euros. MMT sees the purchase of government bonds by the central bank as an asset swap. Government bonds purchased by the ECB, for instance, are not paid off.[15] Government access to central bank financing in the eurozone is therefore limited (Table 1, row 4), at least with the standard rules in place. Since 2020, the general escape clause of the Stability and Growth Pact has been activated and the ECB has initiated its pandemic emergency purchase programme (PEPP), ensuring enough demand for government bonds so that investors perceive them as risk-free.[16] This means that the national governments are free to spend what they think appropriate until the escape clause is deactivated. Public debt sustainability is a political, not an economic issue (Table 1, row 2). This is most clearly visible when looking at Greece, which had a public debt-to-GDP ratio of 130% in the early 2010s when it ran out of money, but has been doing well in 2021 with a public debt-to-GDP ratio of more than 200%.

Government bonds are issued to satisfy eurozone rules (Table 1, row 3). Since they provide a risk-free asset, at least in good times, the bonds are also used as a means to stabilise the interest rate at some positive level. When a central bank buys a government bond from a bank, it just marks up the bank's account. A government bond in the possession of the central bank will lead to an interest payment from the Treasury to the central bank. Since this payment increases the central bank's profits and those are usually transferred to the Treasury's account, it is up for discussion whether government bonds held by a central bank should be counted towards public debt.

Public debt equals the money that a government has spent and not yet collected back in taxes. This is something fundamentally different from a private borrower with debt. The private borrower would have to make a payment to rid herself of debt. The government cannot do that – its payments cause the public debt. Actually, taxpayers would have to make payments in order for public debt to come down towards zero. Therefore, government bonds held by the central bank or households do not constitute a debt that has to be redeemed by the government.

## Mainstream, MMT and real MMT: Economic policy and the table

Drumetz and Pfister (2021a, 357) correctly describe the MMT view on crowding out:

> The crowding-out effect on private spending does not exist in MMT because expansionary fiscal policy is supposed to lower interest rates by providing liquidity to banks rather than raising them by crowding-out the private demand for debt financing.

14 This is only logical, since reserves are basically a tax credit. Neither the central bank nor Treasury make payments to the State – they are part of the state.
15 Interest from bond holdings of the ECB is distributed to the treasuries of the eurozone member states, weighted by the capital key.
16 I have argued for such a programme since the publication of my book in 2014 (in German; Ehnts, 2014).

Most central banks intervene in the money market automatically to ensure that the interest rate does not fall (rise) when the government spends (receives tax revenue). This means that there is no financial crowding-out – the government spends reserves that are created anew (and not taken from some pre-existing pot of savings, like the loanable funds theory implies) and the interest rate does not change.[17] This, however, does not mean that government spending could not potentially crowd out private spending. If a federal government takes over a part of the organisation of an economy, say health care provision or public education, then obviously private sector firms would be crowded out. MMT does not say that the government is better (more efficient or more effective) than the private sector. MMT simply highlights the fact that resources used for social welfare have opportunity costs since they are not available for alternative private (or public) sector uses (Table 1, row 14).

MMT helps us to understand what the monetary system is. It is in place so that the government can provide itself with the resources and workers it needs to do its job, which is to fulfil its public purpose (Ehnts and Höfgen, 2019). It is important to note that the government can only buy what its citizens are willing and able to sell. This means that a government should be interested in having an educated and productive workforce with plenty of skills providing the government with a higher output (Table 1, row 13). Whether those with higher skills also (do or should) receive a higher income is an empirical question and cannot be answered by MMT. The goal of the economy is to provide us with the goods and services that we need. Public purpose can be served by the private sector as well as the public sector.

Another mainstream view is that the economy should be competitive (Table 1, row 12). From a MMT perspective, this is mistaken. The economy should be targeting full employment and price stability. A competitive economy might provide these, but rather by chance and not through macroeconomic policy. If a competitive economy is one in which exports are higher than imports, then the most competitive economy that we can think of is one in which all value added is exported. This means that wages and domestic consumption are zero and all of national income is in the hands of capital owners – hardly a promising target for a modern society. The way this undesirable situation would be achieved is through falling wages (given some exchange rate). The further wages fall, the higher net exports will rise. This used to be called mercantilist policy or beggar-thy-neighbour policy. A neo-mercantilist policy might be successful, as the case of Germany seems to show.

So, what about macroeconomic policy? In mainstream economics, monetary policy has a role to play to stabilise the economy (Table 1, row 7). It is assumed that an increase (decrease) of the central bank's main interest rate will lead to a decrease (increase) in private investment. This view has lost its credibility after almost a decade of zero and negative interest rates and lackluster private investment. MMT and the mainstream agree that an increase of the interest rate might, after some time lag, cause a collapse in private investment that is big enough to bring down wage growth and, with it, inflation. However, almost nobody believes that a fall in the interest rate would bring about a recovery with rising private investment. MMT recognises that changes in aggregate demand matter for private investment. The Biden administration's actions are consistent with this view. Government spending creates, dollar for dollar, private sector income. If firms need to invest before they can sell to the government, then they will do it as long as expected positive profits result. The nominal interest rate is of secondary importance, if at all.

The mainstream view is that the interest rate is and should be the main policy instrument of monetary policy. MMT disagrees. Fighting inflation by creating unemployment through a rise in the interest rate might work, but in the long run it is a socially damaging policy. After four decades, this kind of monetary policy has left most of the Western economies, and the eurozone especially, with high rates of unemployment and high levels of inequality. The eurozone's rate of unemployment has never been below 7%, which is high compared to other developed countries. Given existing technology, working hours and physical capital, a consistent lack of government spending has caused aggregate demand to fall short of what is required for full employment. Mario Draghi, who understands this, has called for more expansionary fiscal policy over his whole reign as ECB president. Monetary policy should support fiscal policy in finding the right level of spending that is consistent with full employment (Table 1, row 7). This means that the ECB should guarantee the national government's liquidity and solvency at all times. Only then can we expect that macroeconomic mindset of policymakers to shift from the austerity mode to a European New Deal mode. With regard to the interest rate, it might make sense to leave it at zero to ensure that nobody earns risk-free rewards or to set it at 2% in order to support the inflation target of the same size.[18]

The question of whether interest rates are set by the central bank or the market has become clearer in the last few years. If the central bank wants, it can steer the overnight

17 This challenges the concept of monetary and fiscal dominance. The central bank can set and control the interest rate and at the same time execute the government's payments, as Fullwiler (2020) shows. Any interest rate the central bank sets is compatible with any level of public debt to GDP.

18 MMT recognises that higher interest rates on Treasury bonds increase aggregate demand.

interest rate and all other interest rates (yields) along the yield curve for government bonds (Mosler and Armstrong, 2019). Japan, while not following Modern Monetary Theory as Wray and Nersisyan (2021) point out, has shown that it is possible to directly target bond yields. This means that markets set interest rates (yields) only to the extent that the central bank lets them (Table 1, row 8).

History has shown that full employment and price stability are compatible. They are not at the opposite ends of a trade-off, as the Phillips curve implies. In many Western European countries, we had both full employment and price stability in the 1960s. Between 1961 and 1966, the German unemployment rate was below 1% for five consecutive years, with inflation rates between 2.4% and 3.3%

According to MMT, both price level and changes in the price level are mostly driven by the behaviour of the state. Due to the monopoly on currency that it enjoys, the state is the only actor in the economic sphere that can pay whatever wages or prices it pleases (Levey, 2021), which sets the price level. When the state pays different prices, the price level changes. This also explains what happens in hyperinflation. The governments of Weimar Germany in the early 1920s and those of Zimbabwe and Venezuela paid higher and higher prices to public employees and also paid more for the currencies, goods and services they procured (Armstrong and Mosler, 2020).

Figure 1 shows the empirical relationship between wages and salaries paid by the US government; it is much tighter than those between monetary aggregates and inflation. Bobeica et al. (2019) in an ECB working paper also find that "labor cost increases will be passed on to prices". Nevertheless, MMT does not deny that there are other influences on prices as well. For instance, a rising oil price can drive up the price level if the rising energy costs are passed on to consumers. Also, monopolistic competition can drive up prices in areas like education and health care. Alternatively, inflation can arise if there is a lack of workers in any given area of the economy, driving up wages there. All of this means that inflation is a symptom of changes in society and not always a "monetary phenomenon" (Table 1, row 9).

An understanding that inflation is not caused by tight labour markets infers that full employment and price stability are possible (Table 1, row 10). Aggregate spending in the economy determines aggregate output, which – given working hours, technology and capital – determines employment. If private spending is not high enough to reach full employment, it is the task of the government to increase spending.[19] After all, it is the tax liabilities that the government

19 Theoretically, a change in tax rates works as well, but not if it reduces taxes mostly for the rich who then save the additional income.

Figure 1

**Government wage growth and inflation in the US**

Percent change from year ago, annual

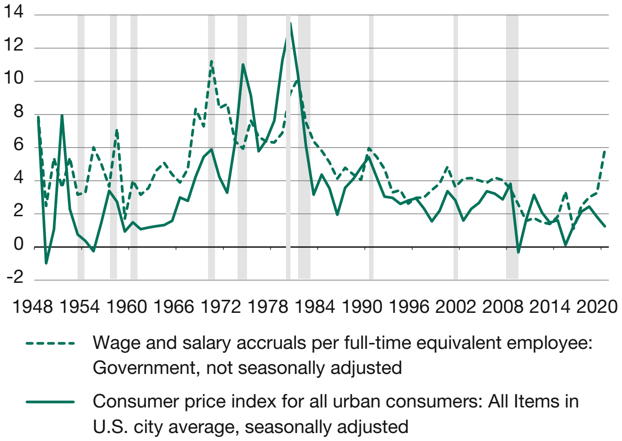

Note: Shaded areas indicate U.S. recessions.

Source: Federal Reserve Bank of St. Louis.

imposes that forces people to look for paid work. Since the government cannot know the future, it is impossible to fine-tune fiscal policy so that full employment results at all times. That is why MMT has suggested the addition of the Job Guarantee (Tcherneva, 2020). In this way, those who can work and want to work always have the option to take on a Job Guarantee job, which would eliminate involuntary unemployment and act as a macroeconomic stabiliser.

The assessment of conventional structural policies from a MMT perspective is open (Table 1, row 11). If conventional structural policies mean imposing hardship on those earning their income mostly through work, there is no reason why this should be a preferred policy. MMT recognises that managing the supply side of the economy and labour relations is important for total productivity and allocation. There is nothing wrong with allocation by the private sector per se. If, however, the results indicate a sub-optimal allocation, then the government should not hesitate to change the rules of the game. This is most important in the context of a Green New Deal (Nersisyan and Wray, 2019).[20]

## Conclusion

Drumetz and Pfister (2021a, 2021b) should be lauded for their intent to engage with MMT. As expected, a cultural shock resulted, as MMT is a falsifiable empirical monetary theory that sets out to explain the real world whereas the mainstream theory sets out from model assumptions and

20 Drumetz and Pfister (2021a, 2021b) make some more points that are worthy of comment which I address in Ehnts (2022).

then moves to the real world. It was the intent of this reply to correct the image of MMT that the authors built up and that is reflected in their Table 1 (also Table 1 of this paper). I have argued that before discussing the macroeconomic implications of MMT (what Drumetz and Pfister call the "meaning") we need to get the balance sheets right. MMT starts with the logic of the payment system (reserve accounting) and then moves on to sectoral balances. Therefore, it provides a discussion of the micro-structure that is absent in most of mainstream macroeconomics. It is at this level that the debate of MMT should start, leaving the question of what to do in terms of macroeconomic policy for later.

Drumetz and Pfister are invited to reply to this paper by engaging with the claims made here. As Table 1 shows, I think that their representation of MMT is flawed and therefore their judgement of MMT is unreliable. To make some progress, I would ask the authors to explain in balance sheets how the French federal government actually spends and/or to refute my balance sheets for the German case. I believe my balance sheet structure shows clearly that the German Bundesbank is a currency issuer and that it creates new reserves every time the German federal government spends. If that is the case everywhere in the eurozone, this would mean that the ECB could solve any problem of fiscal sustainability by making the PEPP permanent, as argued by Ehnts and Paetz (2021). The question of how much governments are allowed to spend is divorced from this issue.

## References

[CR1] Armstrong, P. and W. Mosler (2019, 24 February), A Discussion of Central Bank Operations and Interest Rate Policy, *Gower Initiative for Modern Monetary Studies*.

[CR2] Armstrong, P. and W. Mosler (2020, 14 November), Weimar Republic Hyperinflation through a Modern Monetary Theory Lens, *Gower Initiative for Modern Monetary Studies*.

[CR3] Beveridge, W. H. (2015) [1944], *Full Employment in a Free Society (Works of William H. Beveridge): A Report*, Routledge.

[CR4] Bloomberg, (2019a), Draghi Says ECB Should Examine New Ideas Like MMT.

[CR5] Bloomberg (2019b), Lagarde says MMT is no panacea but may help fight deflation.

[CR6] Bobeica, E., M. Ciccarelli and I. Vansteenkiste (2019), The link between labor cost and price inflation in the euro area, *ECB Working Paper Series*, 2235.

[CR7] Bundesbank (2017), The role of banks, non-banks and the central bank in the money creation process, *Deutsche Bundesbank Monthly Report*, April 2017, 13–33.

[CR8] Carrión Álvarez M, Ehnts D (2016). Samuelson and Davidson on ergodicity: A reformulation. Journal of Post Keynesian Economics.

[CR9] Drumetz F, Pfister C (2021). Modern Monetary Theory: A Wrong Compass for Decision-Making. Intereconomics.

[CR10] Drumetz, F. and C. Pfister (2021b), The Meaning of MMT, *Banque de France Working Paper*, 833.

[CR11] ECB (2021), How quantitative easing works, https://www.ecb.europa.eu/explainers/show-me/html/app_infographic.en.html (5 January 2022).

[CR12] Ehnts D (2014). A simple macroeconomic model of a currency union with endogenous money and saving-investment imbalances. International Journal of Pluralism and Economics Education.

[CR13] Ehnts, D. (2016), *Modern Monetary Theory and European macroeconomics*, Routledge.

[CR14] Ehnts, D. (2019, 4 June), Modern monetary theory: a simple macroeconomic model, *Social Europe*.

[CR15] Ehnts, D. (2020a), The fiscal-monetary nexus of Germany, *IPE Working Paper*, 138/2020.

[CR16] Ehnts D (2020). The unexpected victory of modern monetary theory and its consequences. International Journal of Pluralism and Economics Education.

[CR17] Ehnts, D. (2022), The meaning of MMT: a reply, *EDI Working Paper Series*, 03.

[CR18] Ehnts D, Höfgen M (2019). Modern Monetary Theory and the Public Purpose. American Review of Political Economy.

[CR19] Ehnts D, Paetz M (2021). COVID-19 and its economic consequences for the Euro Area. Eurasian Economic Review.

[CR20] Ehnts, D. and A. Voldsgaard (2020), A Paradigm Lost, a Paradigm Regained — A Reply to Druedahl on Modern Monetary Theory.

[CR21] Fullwiler S (2020). When the Interest Rate on the National Debt Is a Policy Variable (and “Printing Money” Does Not Apply). Public Budgeting & Finance.

[CR22] Godley, W. and M. Lavoie (2006), *Monetary Economics: An Integrated Approach to Credit, Money, Income, Production and Wealth*, Palgrave Macmillan.

[CR23] Greitens, J. (2019), Karl Helfferich und Rudolf Hilferding über Georg Friedrich Knapps “Staatliche Theorie des Geldes”: Geldtheorien zur Zeit der Hyperinflation von 1923, *IBF Paper Series*, 04–19.

[CR24] Ihrig, J., G. C. Weinbach and S. A. Wolla (2021), Teaching the Linkage Between Banks and the Fed: R.I.P. Money Multiplier, *Page One Economics Newsletter*, Federal Reserve Bank of St. Louis.

[CR25] Kelton S (2000). Do Taxes and Bonds Finance Government Spending?. Journal of Economic Issues.

[CR26] Kelton, S. (2019, 4 March), The Clock Runs Down on Mainstream Keynesianism, *Bloomberg Opinion*.

[CR27] Kelton, S. (2020), *The Deficit Myth: Modern Monetary Theory and How to Build a Better Economy*, John Murray.

[CR28] Krugman, P. (2021, 21 May), Krugman Wonks Out: What We Talk About When We Talk About Money, *New York Times*.

[CR29] Lerner A (1947). Money as a Creature of the State. The American Economic Review.

[CR30] Levey, S. (2021), Modeling Monopoly Money: Government as the Source of the Price Level and Unemployment, *Levy Economics Institute Working Paper*, 992.

[CR31] Mackintosh, J. (2021, 21 November), Modern Monetary Theory Isn’t the Future. It’s Here Now, *The Wall Street Journal*.

[CR32] McLeay M, Radia A, Thomas R (2014). Money creation in the modern economy. BoE Quarterly Bulletin.

[CR33] Mitchell, W. (2019), A response to Greg Mankiw — Part 1, *Bill Mitchell — Modern Monetary Theory Blog*.

[CR34] Mitchell, W., M. Watt and L. R. Wray (2019), *Macroeconomics*, Red Globe Press.

[CR35] Mosler, W. (1995), Soft Currency Economics.

[CR36] Mosler W (1997). Full Employment and Price Stability. Journal of Post Keynesian Economics.

[CR37] Nersisyan, Y. and L. R. Wray (2019), How to Pay for the Green New Deal, *Levy Economics Institute Working Paper*, 931.

[CR38] New York Times (2021), Modern Monetary Theory Has a New Friend in Congress, https://www.nytimes.com/2021/09/01/opinion/mmt-johnyarmuth.html (5 January 2022).

[CR39] Reuters (2020, 19 November), ECB can’t go bankrupt even it suffers losses.

[CR40] Sheard, P. (2013), Repeat After Me: Banks Cannot and Do Not Lend Out Reserves, *S&P Ratings Direct Economic Research*, 1–15.

[CR41] Tcherneva P (2002). Monopoly Money: The State as a Price Setter. Oeconomicus.

[CR42] Tcherneva, P. (2020), *The Case for a Job Guarantee*, Polity Press.

[CR43] Tymoigne, É. (2014), Modern Money Theory and interrelations between the treasury and the central bank: The case of the United States, *Levy Economics Institute Working Paper*, 788.

[CR44] Wray, L. R. and Y. Nersisyan (2021), Has Japan Been Following Modern Money Theory Without Recognizing It? No! And Yes, *Levy Economics Institute Working Paper*, 985.

